# High glucose impairs autophagy and mitochondrial homeostasis in human dental pulp cells

**DOI:** 10.1038/s41598-026-54142-7

**Published:** 2026-05-26

**Authors:** Zijiang Yang, Xiangzi Zhang

**Affiliations:** 1 Department of Stomatology, The Affiliated Hospital of Yanbian University (Yanbian Hospital), Yanji, Jilin China; 2 Department of Stomatology, College of Medicine, Yanbian University, Yanji, Jilin China

**Keywords:** High glucose, Human dental pulp cells, Autophagy, Mitochondrial homeostasis, AMPK/mTOR signaling, Biochemistry, Cell biology, Diseases, Endocrinology, Molecular biology

## Abstract

This study aimed to investigate the effects of high-glucose exposure on human dental pulp cells and to explore associated changes in autophagy, mitochondrial homeostasis, and AMPK/mTOR signaling. Human dental pulp cells were cultured in vitro and assigned to a control group, a 5 mmol/L low-glucose group, and a 35 mmol/L high-glucose group. Cell viability was assessed using the CCK-8 assay at 24, 48, 72, and 96 h. The expression levels of p-AMPKα, p-mTOR, SQSTM1/p62, LC3B, and β-actin were detected by western blotting. Intracellular reactive oxygen species were measured using DCFH-DA staining, and cellular glutathione and ATP levels were evaluated using assay kits. Compared with the low-glucose group, high-glucose exposure increased p62 expression, reduced LC3B expression, decreased AMPKα phosphorylation, and increased mTOR phosphorylation. High-glucose exposure also increased intracellular reactive oxygen species levels, reduced glutathione content, and decreased ATP levels. These findings suggest that high-glucose exposure impairs autophagy-related activity and disrupts mitochondrial homeostasis in human dental pulp cells, accompanied by changes in AMPK/mTOR signaling.

**Supplementary Information:**

The online version contains supplementary material available at 10.1038/s41598-026-54142-7.

## Introduction

Dental pulp diseases are among the most common oral pathologies and are a major cause of tooth pain and tooth loss. The development of these diseases is complex, involving multiple pathological processes. Dental caries remains highly prevalent and is an important cause of pulpitis and periapical disease when lesions progress into deep dentin^1^. The occurrence of pulp diseases is influenced by various factors, including oral hygiene practices, dietary habits, smoking, aging, and metabolic disorders. Diabetic patients show a higher risk of pulp-related pathological changes, with distinct clinical and histological characteristics compared with non-diabetic individuals^2,3^. High-glucose conditions may contribute to dental pulp alterations through oxidative stress-related processes, including vascular wall thickening, fibrosis, calcification, localized necrosis, compromised blood supply, neural changes, and reduced regenerative potential^2^.

Treating pulp diseases in diabetic patients presents many challenges in clinical practice. Hyperglycemia-induced pulp tissue fibrosis and vascular lesions impair its self-repair capacity, which may reduce the effectiveness of conventional pulp preservation therapies, such as vital pulp therapy. The pulp tissue of diabetic patients frequently shows calcification and necrosis, making root canal treatment more difficult and increasing the risk of treatment failure^2^. When treating pulp diseases in diabetic patients, clinicians should consider glycemic control, the pathological condition of the pulp tissue, and treatment feasibility. Several approaches have been explored to reduce hyperglycemia-associated pathological changes in pulp tissue, including antioxidant, anti-inflammatory, and dental pulp stem cell-based strategies. Antioxidants such as N-acetylcysteine and vitamin C may help reduce oxidative stress caused by high-glucose conditions, whereas anti-inflammatory agents may alleviate inflammatory responses in dental pulp tissue. In vitro expansion and transplantation of dental pulp stem cells may also provide potential strategies for pulp tissue repair^4^. However, the cellular mechanisms by which high-glucose conditions affect dental pulp cell homeostasis remain incompletely understood.

Recent studies have increasingly focused on the role of autophagy in cellular self-protection against pulp diseases. Autophagy maintains intracellular stability by degrading damaged organelles and proteins, playing a critical role in inflammation, infection, and tissue repair^5–7^. Although autophagy has been studied in pulpitis and apical periodontitis, its specific regulation in dental pulp cells under hyperglycemic conditions remains incompletely understood. Given the increasing clinical relevance of diabetes-related oral complications, it is important to clarify how high-glucose exposure affects autophagy and mitochondrial homeostasis in dental pulp cells. Therefore, this study aimed to investigate the effects of high-glucose exposure on autophagy and mitochondrial homeostasis in human dental pulp cells and to explore associated changes in AMPK/mTOR signaling. These findings may provide preliminary cellular evidence for understanding high-glucose-related pulp pathology in the context of diabetes.

## Materials and methods

### Extracted tooth samples

Healthy permanent teeth were collected from patients requiring extraction for routine clinical orthodontic reasons unrelated to this study (*n* = 8 premolars and *n* = 6 third molars). No teeth were extracted solely for research purposes. All participating patients were fully informed of the study’s purpose, content, and methodology and provided written informed consent for the use of their samples and data. This study was approved by the Ethics Committee of Yanbian University Affiliated Hospital (Yanbian Hospital), approval number 2025407. Research procedures adhered to the principles of the Declaration of Helsinki and complied with relevant laws and regulations of China’s Good Clinical Practice (GCP) (Table 1).

**Table 1:** Cell viability values of human dental pulp cells in the low-glucose and high-glucose groups.

Group	24 h	48 h	72 h	96 h
LG	99.44 ± 0.45	97.76 ± 2.30	98.05 ± 1.01	94.35 ± 1.93
HG	76.96 ± 1.33	75.06 ± 3.75	75.71 ± 1.28	74.55 ± 2.58

Data are presented as the mean ± SD (*n* = 3). LG, low-glucose group; HG, high-glucose group.

### Experimental materials

RIPA lysis buffer (Cat. No. P0013B), PMSF (Cat. No. ST506), primary and secondary antibody stripping solution (Cat. No. P0025), 30% Acr-Bis (29:1; Cat. No. ST003), SDS-PAGE protein loading buffer (Cat. No. P0015), ECL luminescent solution (Cat. No. P0018), BCA protein concentration determination kit (Cat. No. P0009), CCK-8 kit, reactive oxygen species detection kit (DCFH-DA kit), adenosine triphosphate (ATP) detection kit, and goat anti-rabbit IgG-HRP (Beyotime Institute of Biotechnology, China, Cat. No. A0208); glutathione assay kit (Sigma-Aldrich, USA); ECL luminescent solution (Millipore, USA, Cat. No. WBKLS0100); Anti-SQSTM1/p62 antibody (Cat. No. ab207305), anti-AMPK alpha 1 (phospho T183) + AMPK alpha 2 (phospho T172) antibody [EPR5683] (Cat. No. ab133448), anti-mTOR (phospho S2481) antibody (Cat. No. ab137133), and LC3B antibody (Cat. No. ab192890) (Abcam, China); β-actin antibody (Zhongshan Golden Bridge Biotechnology, China, Cat. No. ab8226); goat anti-mouse secondary antibody (Cat. No. 31430) and goat anti-rabbit secondary antibody (Cat. No. 31460) (Thermo, USA); pre-stained protein molecular weight marker (Fermentas, Canada, Cat. No. 26616); PVDF membrane (Millipore, USA, Cat. No. IPVH00010); skimmed milk powder (Yili, China, Cat. No. Q/NYLB 0039 S).

### Experimental methods

#### Dental pulp cell culture and grouping

**Fig. 1:**
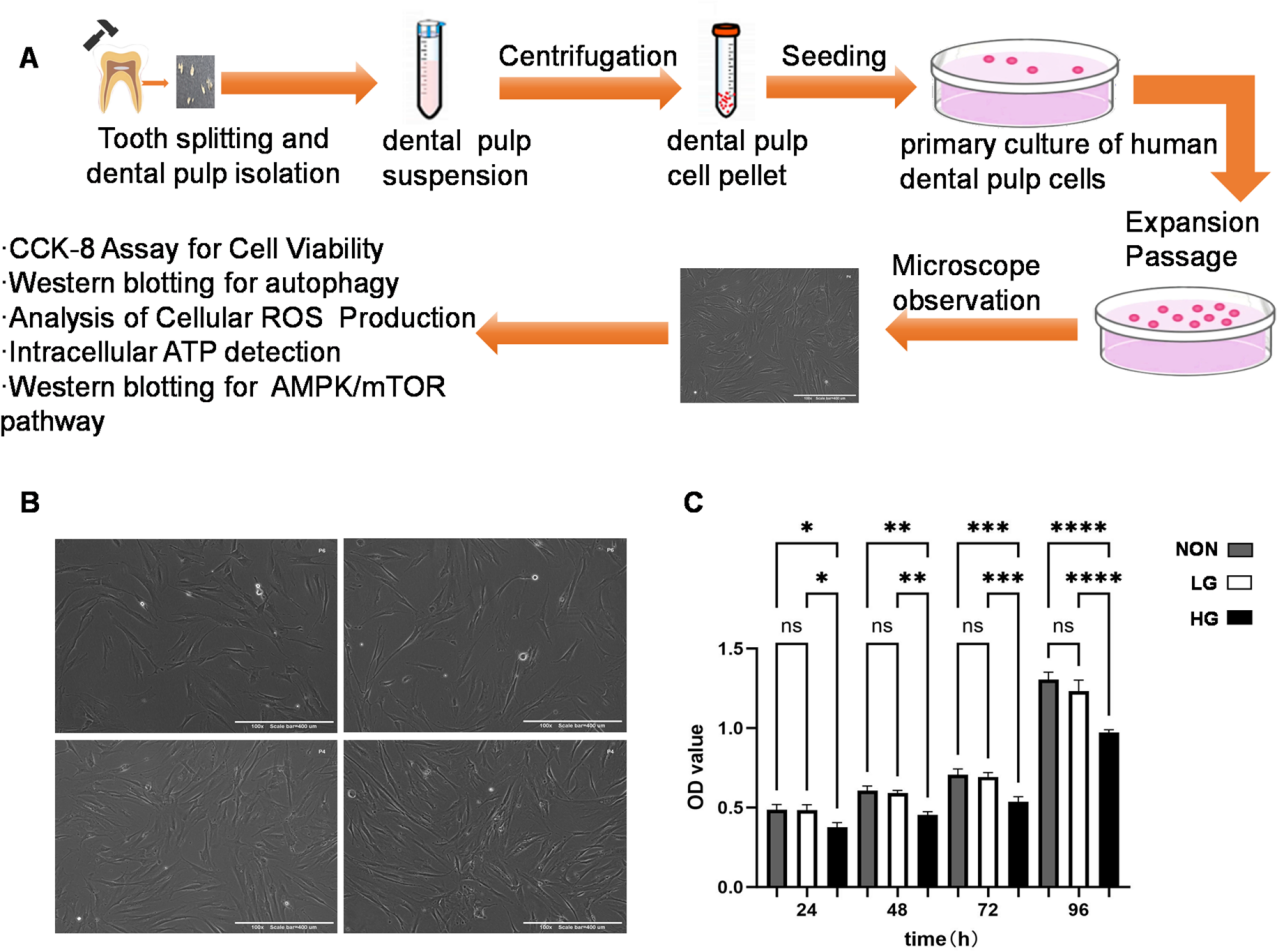
Isolation, identification, and viability analysis of human dental pulp cells under high-glucose conditions. **(A)** Experimental workflow. **(B)** Representative microscopic images of human dental pulp cells cultured and passaged to generations 4–6. Cells showed a typical fibroblast-like spindle morphology under an inverted microscope at 100× magnification. **(C)** CCK-8 assay showing optical density values of human dental pulp cells in the control, low-glucose, and high-glucose groups at 24, 48, 72, and 96 h. Cell viability remained relatively stable in the low-glucose group, whereas it was significantly reduced in the high-glucose group over time. Data are presented as the mean ± SD. n.s., not significant. *P < 0.05, **P < 0.01, ***P < 0.001, and ****P < 0.0001.

Freshly extracted healthy premolars and caries-free third molars were collected for dental pulp cell culture. The teeth were placed in a centrifuge tube containing α-MEM medium supplemented with 10% (v/v) fetal bovine serum (FBS) and kept in an ice box. Under sterile conditions in a laminar flow hood, the teeth were washed with PBS, and the periodontal ligament was carefully scraped off. The cleaned teeth were then placed in a sterile bag and cracked open using a hammer to extract the pulp tissue, which was transferred into an EP tube containing PBS and cut into small fragments with scissors. After centrifuging at 1000 rpm for 5 min, the supernatant was discarded, and 1 mL of collagenase was added. The mixture was incubated at 37 °C with 5% CO₂ for 45 min, with shaking every 5 min. It was then centrifuged again at 1000 rpm for 5 min, the supernatant discarded, and the pellet washed with medium containing 10% FBS, followed by centrifugation. This washing and centrifugation step was repeated three times before discarding the final supernatant. The tissue fragments were aspirated with a pipette and placed into a 25 mL culture flask containing about 2 mL of medium with 20% FBS. Approximately 500 µL of medium with 20% FBS was added to the EP tube to resuspend any remaining cells, which were also transferred to the culture flask. The flask was incubated at 37 °C with 5% CO_2_. Additional medium was added as needed once cells began migrating out from the tissue pieces. The morphology and growth of the dental pulp cells were monitored under a microscope. When the cells reached approximately 80% confluence, they were digested with trypsin and passaged for subsequent experiments. Cells from passages 3–5 were used for the assays. The experimental workflow is illustrated in Fig. 1A.

#### Establishment of the high-glucose model and CCK-8 assay

The experimental groups included a control group, a low-glucose group (LG), and a high-glucose group (HG) at 24, 48, 72, and 96 h. The glucose concentration was 5 mmol/L in the LG group and 35 mmol/L in the HG group. The control group was maintained under standard culture conditions. After normal cell culture for 24 h, the medium was replaced with fresh medium according to the experimental grouping. Cell viability was measured after 24, 48, 72, and 96 h of treatment following the kit instructions. Each experiment was repeated three times.

#### Western blot analysis

Collected cell pellets were lysed on ice for 30 min with lysis buffer, followed by centrifugation for 20 min. The supernatant was aliquoted into 1.5 mL centrifuge tubes, with a portion used for protein concentration determination. Protein standard solution was diluted, and 1 µL of each sample was added to sample wells and made up to 20 µL with double-distilled water. BCA working solution was prepared, and the absorbance at OD560 was measured for standard solutions of various concentrations and protein samples. A standard curve was plotted to calculate sample concentrations. Equal amounts of protein were separated by SDS-PAGE and transferred onto PVDF membranes. After blocking, membranes were incubated with primary antibodies against p62, LC3B, p-AMPKα, p-mTOR, and β-actin, followed by incubation with HRP-conjugated secondary antibodies. Protein bands were visualized using ECL reagents and quantified relative to β-actin. All primary and secondary antibodies were used according to the manufacturers’ recommended dilutions.

#### DCFH-DA assay

Cellular ROS production was measured using DCFH-DA staining. Cells were seeded into 96-well black plates at a density of 1 × 10^4^ cells/well in standard culture medium and allowed to adhere overnight. Cells were then cultured under LG or HG conditions for 96 h. After treatment, cells were incubated with 100 µM DCFH-DA, and the fluorescence intensity of dichlorofluorescein (DCF) was measured using a microplate reader (Synergy H1, BioTek) at an excitation wavelength of 485 nm and an emission wavelength of 535 nm. ROS levels were quantified based on DCF fluorescence intensity.

#### GSH assay

Reduced glutathione (GSH) was measured using a GSH assay kit (Sigma-Aldrich) according to the manufacturer’s instructions. Cells were cultured under LG and HG conditions for 96 h, then scraped, lysed with lysis buffer, and centrifuged. The supernatant was transferred to a black microplate, followed by the addition of glutathione S-transferase (GST) and monochlorobimane substrate. Fluorescence intensity was measured at an excitation wavelength of 390 nm and an emission wavelength of 478 nm. GSH levels were normalized to total protein concentration. Protein concentration was determined using a BCA protein assay kit (Beyotime, China) according to the manufacturer’s instructions.

#### Intracellular ATP detection

Intracellular ATP levels were measured using an ATP assay kit (Beyotime) according to the manufacturer’s protocol. Human dental pulp cells were lysed, and the supernatant was collected after centrifugation at 4 °C. ATP content was quantified using a multimode microplate reader (BioTek).

### Statistical analysis

SPSS 27.0 software was used for statistical analysis. Data conforming to a normal distribution were expressed as the mean ± standard deviation (mean ± SD). One-way analysis of variance (one-way ANOVA) was used for comparisons among groups, and the least significant difference (LSD) test was used for pairwise comparisons. A two-tailed test was performed, with a significance level of α = 0.05. Graphs were generated using GraphPad Prism 9.5. A *P* value < 0.05 was considered statistically significant.

## Results

### Isolation and identification of dental pulp cells

Human dental pulp cells were successfully cultured in vitro using the tissue block enzyme digestion method. The cells adhered well and were passaged to generations 4–6. The cells showed a typical fibroblast-like spindle morphology under an inverted microscope at 100× magnification (Fig. 1B). Cells were passaged when they reached approximately 80% confluence.

### CCK-8 assay for cell viability

To evaluate the effect of high-glucose conditions on dental pulp cell viability, we performed the CCK-8 assay at 24, 48, 72, and 96 h under low-glucose and high-glucose conditions (Fig. 1C). Dental pulp cells from passages 3–5 were used for the assay. Cell viability remained relatively stable in the low-glucose group throughout the experiment. In contrast, the high-glucose group showed a time-dependent decrease in cell viability. At 96 h, cell viability in the high-glucose group was significantly lower than that in the low-glucose group (*P* < 0.001), indicating that high-glucose exposure reduced dental pulp cell viability.

### High-glucose exposure is associated with reduced autophagy-related activity in dental pulp cells

**Fig. 2:**
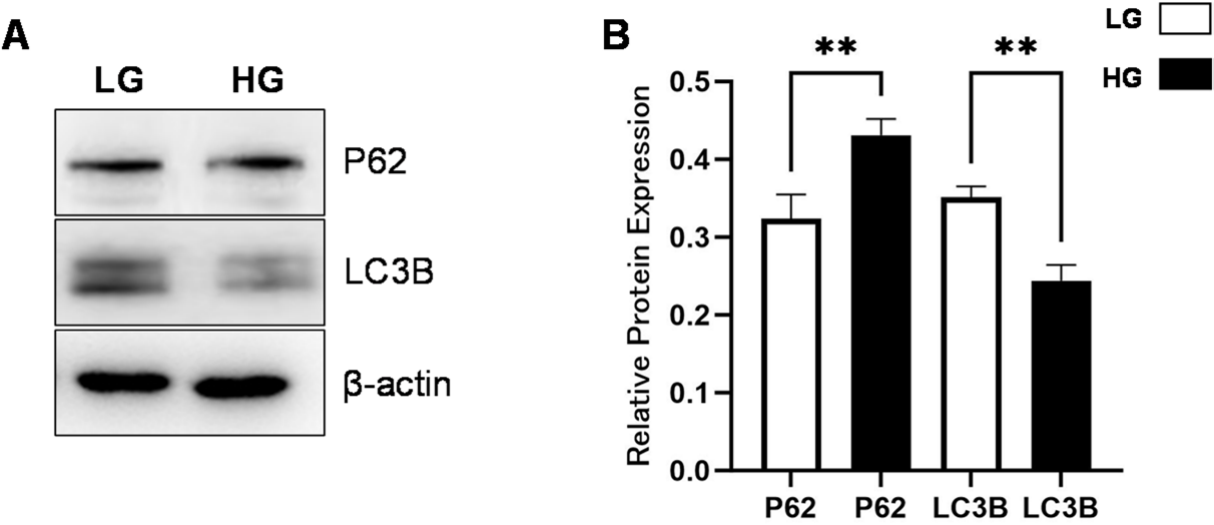
High-glucose exposure altered autophagy-related protein expression in human dental pulp cells. **(A)** Representative western blot images of p62, LC3B, and β-actin expression in the low-glucose and high-glucose groups. **(B)** Quantitative analysis showed increased p62 expression and decreased LC3B expression in the high-glucose group compared with the low-glucose group. Data are presented as the mean ± SD. LG, low-glucose group; HG, high-glucose group. **P < 0.01 compared with the low-glucose group.

To evaluate the effect of high-glucose exposure on autophagy-related proteins in dental pulp cells, cells were treated under low-glucose and high-glucose conditions for 96 h. Western blot analysis was conducted to detect the expression levels of p62 and LC3B. As shown in Fig. 2A and B, compared with the low-glucose group, the high-glucose group showed increased p62 expression (*P* < 0.01) and decreased LC3B expression (*P* < 0.01). These results suggest that high-glucose exposure was associated with reduced autophagy-related activity in dental pulp cells.

### High-glucose exposure increases ROS production and reduces GSH and ATP levels in dental pulp cells

**Fig. 3:**
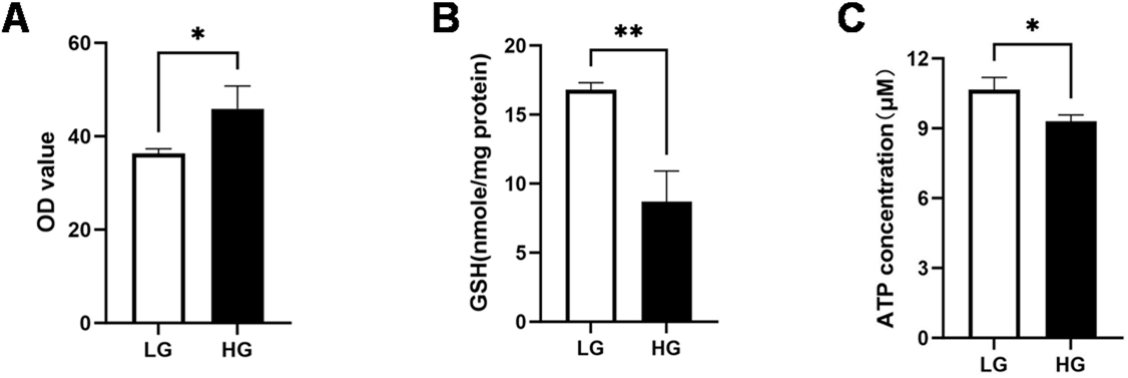
High-glucose exposure increased ROS production and reduced GSH and ATP levels in human dental pulp cells. **(A)** Quantitative analysis of intracellular ROS levels based on DCF fluorescence intensity in the low-glucose and high-glucose groups. **(B)** Quantitative analysis of GSH levels in the low-glucose and high-glucose groups. **(C)** Quantitative analysis of intracellular ATP concentration in the low-glucose and high-glucose groups. Data are presented as the mean ± SD. LG, low-glucose group; HG, high-glucose group. *P < 0.05 and **P < 0.01 compared with the low-glucose group.

The overall intracellular ROS levels were quantified based on DCF fluorescence intensity, as shown in Fig. 3A. Compared with the low-glucose group, the high-glucose group showed significantly increased DCF fluorescence intensity (*P* < 0.05), indicating increased ROS production. GSH levels were significantly lower in the high-glucose group than in the low-glucose group (*P* < 0.01; Fig. 3B), suggesting reduced antioxidant capacity. Intracellular ATP levels were also significantly reduced in the high-glucose group compared with the low-glucose group (*P* < 0.05; Fig. 3C), indicating reduced cellular energy availability under high-glucose conditions.

### High-glucose exposure is accompanied by altered AMPK/mTOR signaling in dental pulp cells

**Fig. 4:**
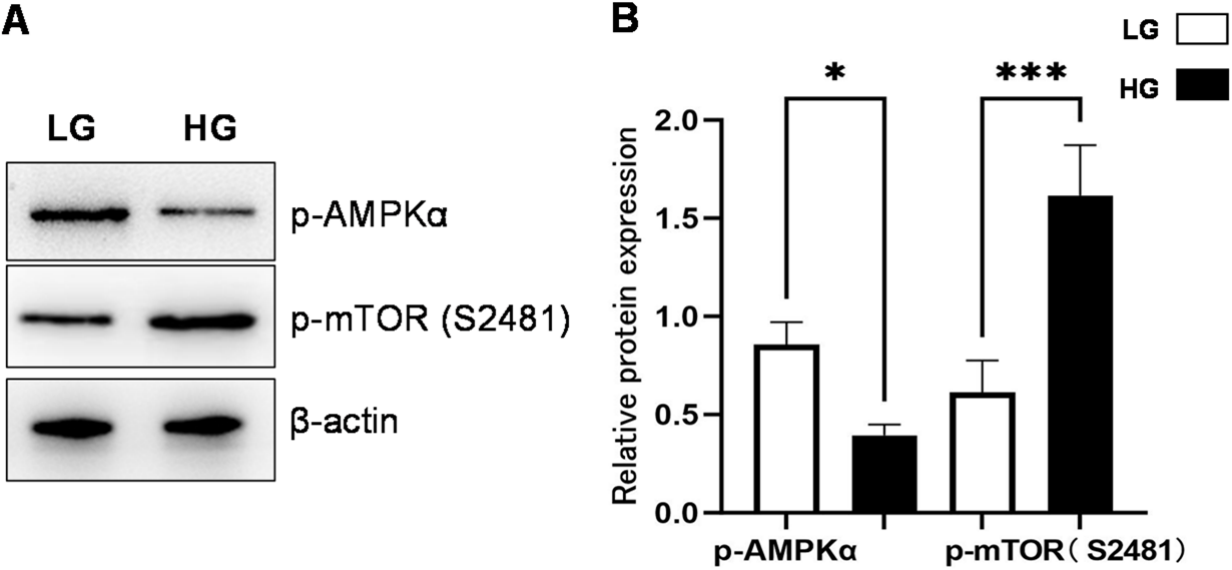
High-glucose exposure was accompanied by altered AMPK/mTOR signaling in human dental pulp cells. **(A)** Representative western blot images of p-AMPKα, p-mTOR, and β-actin expression in the low-glucose and high-glucose groups. **(B)** Quantitative analysis showed decreased p-AMPKα expression and increased p-mTOR expression in the high-glucose group compared with the low-glucose group. Data are presented as the mean ± SD. LG, low-glucose group; HG, high-glucose group. *P < 0.05 and ***P < 0.001 compared with the low-glucose group.

To explore signaling changes associated with autophagy under high-glucose conditions, western blot analysis was performed to detect phosphorylated AMPKα (p-AMPKα) and phosphorylated mTOR (p-mTOR) expression (Fig. 4A, B). Compared with the low-glucose group, the high-glucose group showed decreased AMPKα phosphorylation and increased mTOR phosphorylation. Together with the observed changes in p62 and LC3B expression, these findings suggest that AMPK/mTOR signaling may be associated with the autophagy-related changes induced by high-glucose exposure.

## Discussion

Based on the present findings, high-glucose exposure was associated with reduced cell viability, altered autophagy-related protein expression, increased ROS production, decreased GSH levels, reduced intracellular ATP levels, and changes in AMPK/mTOR signaling in human dental pulp cells. These changes suggest that high-glucose conditions may disrupt dental pulp cell homeostasis through combined effects on oxidative stress, energy metabolism, and autophagy-related cellular responses. Importantly, the present study should be interpreted as an exploratory cellular study rather than definitive evidence of pathway-dependent causality.

The reduction in cell viability observed under high-glucose conditions is consistent with previous studies showing that high glucose can impair the proliferation and function of dental pulp cells and other cell types^8,9^. In this study, the inhibitory effect became more evident with prolonged exposure and was most pronounced after 96 h of treatment with 35 mmol/L glucose. This time-dependent reduction suggests that sustained high-glucose stress may progressively compromise the adaptive capacity of dental pulp cells. Because dental pulp tissue has limited collateral circulation and is enclosed within hard tissue, prolonged cellular stress may be particularly relevant to the reduced reparative potential observed in diabetic pulp pathology. However, the CCK-8 assay reflects cell viability and metabolic activity rather than a specific mode of cell death; therefore, additional assays are required to distinguish whether high-glucose exposure primarily affects proliferation, apoptosis, necrosis, or other forms of cell injury.

Autophagy is an important cellular process that maintains intracellular homeostasis by degrading damaged organelles and proteins and has been implicated in inflammation, infection, tissue repair, pulpitis, and periapical disease^5–7,10,11^. Previous studies have suggested that autophagy may help dental pulp cells adapt to inflammatory or metabolic stress by limiting excessive inflammation, apoptosis, and tissue injury^7,12,13^. In the present study, high-glucose exposure increased p62 expression and decreased LC3B expression in human dental pulp cells, suggesting reduced autophagy-related activity under high-glucose conditions. These findings are also consistent with previous reports showing that high-glucose conditions can affect dental pulp cell function and autophagy-related cellular responses^14,15^. The simultaneous increase in p62 and decrease in LC3B may indicate a shift in autophagy-related protein homeostasis under high-glucose stress. However, because p62 and LC3B reflect steady-state autophagy-related protein levels rather than dynamic autophagic flux, these markers alone cannot determine whether high glucose suppresses autophagy induction, impairs autophagosome formation, or alters lysosomal degradation. Further studies using lysosomal inhibitors and autophagic flux assays are therefore required.

Oxidative stress and disruption of mitochondrial homeostasis are closely linked to metabolic stress under high-glucose conditions. In this study, high-glucose exposure increased ROS production, reduced GSH levels, and decreased ATP levels in human dental pulp cells. These results suggest that high-glucose exposure may create a cellular state characterized by increased oxidative burden, weakened antioxidant defense, and reduced energy availability. Since GSH is an important intracellular antioxidant and ATP levels reflect cellular energy status, the combined reduction in GSH and ATP may indicate that dental pulp cells fail to fully compensate for high-glucose-induced oxidative and metabolic stress. This imbalance may further aggravate cellular vulnerability, particularly in cells exposed to sustained hyperglycemic conditions.

The relationship between mitochondrial homeostasis and autophagy is likely to be functionally important in this context. Mitochondrial stress can increase ROS production and disturb cellular energy metabolism, while autophagy-related pathways contribute to the clearance of damaged cellular components and the maintenance of organelle quality control^16–19^. In the present study, reduced ATP levels and increased ROS levels occurred alongside altered p62 and LC3B expression, suggesting a possible link between mitochondrial stress and autophagy-related changes under high-glucose exposure. Nevertheless, this interpretation should remain cautious. The present study did not directly assess mitochondrial membrane potential, mitochondrial morphology, respiratory function, mitochondrial dynamics, or mitophagic flux. Therefore, mitochondrial homeostasis in this study should be interpreted based on ROS, GSH, and ATP-related indicators rather than as a complete characterization of mitochondrial injury.

AMPK/mTOR signaling is an important nutrient- and energy-sensing pathway involved in autophagy regulation^19–21^. In the present study, high-glucose exposure was accompanied by decreased AMPKα phosphorylation and increased mTOR phosphorylation. This pattern is compatible with reduced energy-sensing activity and enhanced mTOR-related signaling, both of which may be associated with reduced autophagy-related activity. Together with the observed changes in p62 and LC3B expression, these findings suggest that AMPK/mTOR signaling may participate in the cellular response of dental pulp cells to high-glucose stress. However, because pathway-specific intervention experiments were not performed, including pharmacological activation or inhibition and genetic modulation of AMPK or mTOR, these results cannot establish direct causality. Therefore, AMPK/mTOR signaling should be considered a potential associated pathway rather than a confirmed mechanistic mediator in the present study.

Several limitations must be acknowledged when interpreting these findings. First, the present study assessed steady-state levels of autophagy-related proteins, but did not include lysosomal inhibition assays or dynamic autophagic flux analysis. Therefore, we cannot definitively distinguish between reduced autophagy induction and impaired lysosomal degradation. Second, although changes in p-AMPKα, p-mTOR, p62, LC3B, ROS, GSH, and ATP were observed under high-glucose conditions, the study did not include pharmacological rescue experiments or genetic modulation of AMPK or mTOR. Thus, the findings should be interpreted as associative rather than definitive mechanistic evidence. Third, although primary human dental pulp cells provide physiological relevance, donor variability and the limited sample size may affect the generalizability of the findings. Future studies using autophagic flux assays, pathway-specific interventions, mitochondrial functional assays, and larger sample sizes are required to further clarify these associations and determine whether AMPK/mTOR signaling directly mediates the observed cellular changes.

In summary, this study suggests that high-glucose exposure is associated with reduced cell viability, impaired autophagy-related activity, increased oxidative stress, weakened antioxidant defense, and disrupted mitochondrial homeostasis in human dental pulp cells. These alterations were accompanied by changes in AMPK/mTOR signaling, suggesting a possible association between this pathway and the cellular stress responses induced by high-glucose exposure. The findings provide preliminary cellular evidence for understanding diabetes-related pulp pathology, but further functional validation is required to establish direct mechanistic causality.

## Supplementary Information

Below is the link to the electronic supplementary material.

Supplementary Material 1

## Data Availability

The datasets used and/or analysed during the current study are available from the corresponding author on reasonable request.

## Abbreviation

HDPCs: Human dental pulp cells
