# Supplementary material for: High glucose impairs autophagy and mitochondrial homeostasis in human dental pulp cells
Source: Sci Rep. 2026 May 26;16:24089. doi: 10.1038/s41598-026-54142-7 (PMC13439202; doi:10.1038/s41598-026-54142-7)
Supplement: Supplementary file 1 [file 41598_2026_54142_MOESM1_ESM.docx]

Figure2：

P62:

LC3B

Ampkα：

mTOR2481:

β-actin:
